# A Cytosolic Protein Kinase STY46 in *Arabidopsis thaliana* Is Involved in Plant Growth and Abiotic Stress Response

**DOI:** 10.3390/plants9010057

**Published:** 2020-01-02

**Authors:** Shaoyun Dong, Fenglan Zhang, Diane M. Beckles

**Affiliations:** 1 Department of Plant Sciences, University of California, One Shields Avenue, Davis, CA 95616, USA; dongshaoyun@caas.cn; 2 College of Agronomy, Inner Mongolia Agricultural University, Hohhot 010019, China; zhangfenglan041105@imau.edu.cn

**Keywords:** abiotic stress, carbon allocation, carbon partitioning, protein kinases

## Abstract

Starch provides plants with carbon and energy during stressful periods; however, relatively few regulators of starch metabolism under stress-induced carbon starvation have been discovered. We studied a protein kinase Ser/Thr/Tyr (STY) 46, identified by gene co-expression network analysis as a potential regulator of the starch starvation response in *Arabidopsis thaliana*. We showed that STY46 was induced by (1) abscisic acid and prolonged darkness, (2) by abiotic stressors, including salinity and osmotic stress, and (3) by conditions associated with carbon starvation. Characterization of STY46 T-DNA knockout mutants indicated that there was functional redundancy among the STY gene family, as these genotypes did not show strong phenotypes. However, Arabidopsis with high levels of STY46 transcripts (OE-25) grew faster at the early seedling stage, had higher photosynthetic rates, and more carbon was stored as protein in the seeds under control conditions. Further, OE-25 source leaf accumulated more sugars under 100 mM NaCl stress, and salinity also accelerated root growth, which is consistent with an adaptive response. Salt-stressed OE-25 partitioned ^14^C towards sugars and amino acids, and away from starch and protein in source leaves. Together, these findings suggested that STY46 may be part of the salinity stress response pathway that utilizes starch during early plant growth.

## 1. Introduction

Many plants experience unfavorable environments during their lifecycle [1]. These environments often alter plants’ ability to assimilate, partition, allocate, and store carbohydrates [2,3,4]. When photosynthetic efficiency is inhibited by adverse conditions, the sugars produced may be insufficient to drive normal growth [5], and if the stressful conditions progress, the cellular sugar content may become exhausted to levels lower than those needed for sustenance [6,7].

Plants have evolved a myriad of coping mechanisms to survive low carbon availability, which often occurs during environmental stress [8,9,10]. Once sugar starvation is detected, signal transduction cascades are activated, which alter gene expression [11], leading to the metabolism of cytosolic and storage proteins [8,12,13,14,15,16]. If starvation persists, structural biomolecules, such as cell wall polysaccharides and proteins are then degraded [17,18]. The sugars produced at the expense of these growth components gradually replace the depleted carbohydrates. If control conditions are restored in a timely fashion, plants may resume growth. These series of events, called the sugar starvation response (SSR), are aimed to acquire the energy necessary for immediate survival until more favorable conditions prevail [19,20].

The SSR is a complex process that is important for plant stress survival, but its regulation is poorly understood [20]. To identify regulators involved in the SSR, a gene co-expression network analysis was performed with public transcriptomic data [21]. Genes within co-expression modules, i.e., that are similarly expressed under different conditions, may share conserved biological functions [22]. Within this network, the transcript of Ser/Thr/Tyr kinase isoform 46 (STY46) connected two large modules, one containing genes involved in amino acids, lipid, cell wall metabolism, and sugar signaling, and the other containing genes associated with transcriptional regulation [21]. Genes that are highly connected to many other genes of the network are defined as hub genes and usually play a key role in the biological system [23]. Therefore, STY46, which is a hub gene in this SSR network [21], was considered as a potential master regulator in SSR.

The STY kinase family has not been extensively studied, but evidence suggests that some isoforms could regulate SSR [21]. Members of the STY kinase family are dual-specificity kinases that possess catalytic Ser/Thr and Tyr domains, and they regulate some plant metabolic and developmental processes through phosphorylation of target proteins [24,25]. For example, STY protein kinases appear to have important roles in ammonium transport in rice seedling roots [26], in storage oil accumulation in Arabidopsis siliques [27], and in stress response and seed development in cucumber and peanuts [28,29,30].

In Arabidopsis, there are 57 STY isoforms grouped into nine subfamilies, but few have been studied [24]. Three STY-like kinases, e.g., STY8, STY17, and STY46, phosphorylate the transit peptides of chloroplast-targeted pre-proteins in cotyledons [25]. Each of the single, double, and triple mutants of STY46 with STY8 and STY17 all showed reduced nuclear-encoded chloroplast proteins, retarded photosynthetic establishment and lower chlorophyll content during the early stages of de-etiolation (greening) [25]. Thus, STY46 influence on chloroplast function could have repercussions for source activity. STY46 was recently shown to be involved in the translocation of some mitochondrial pre-proteins, suggesting a potential role in plant energy generation [31].

When all of these data are considered, it is reasonable to hypothesize that STY46 could be a potential regulator of the SSR in Arabidopsis through changes in source leaf metabolism. This may be due to the regulation of chloroplast imported proteins, including those needed for photosynthesis and carbon fixation. STY46 could therefore affect carbon, and perhaps energy availability, and could have a role in response to sugar starvation. If so, transgenic lines with different levels of STY46 transcripts would be expected to show differential growth and response to environmental stresses that lead to sugar starvation. Therefore, the objective of this study is to characterize transgenic lines with differential levels of STY46 under normal and abiotic stress conditions to test if STY46 affects growth and abiotic stress response. This work could inform on mechanisms integrating carbon use in plant response and adaptation to adverse environmental stress and could enable better engineering strategies to develop crops that show more robust growth under abiotic stress.

## 2. Results

### 2.1. In Silico Sequence and Expression Analysis of STY46

Genevestigator^®^ analyses [32] of *A. thaliana* STY46 suggested that STY46 is expressed at low levels at distinct stages of the lifecycle, but that transcripts are stimulated by stress. STY46 expression was highest in germinating seeds, seedlings, and young rosettes (Supplementary Figure S1A). Spatially, expression of STY46 was highest in sperm cells, anthers, embryos, and endosperm (Supplementary Figure S1B). STY46 transcript was affected by hormones, light intensity and quality, nutrient status, photoperiodicity, and some abiotic stresses. Further, STY46 transcript was up-regulated by nitrate deficiency (3.5-fold), salicylic acid (3–5-fold), dark (4-fold), extended dark (4–6-fold), and hypoxia (3–14-fold), but was down-regulated by glucose (37-fold), sucrose (7–9-fold), and cold (3–12-fold) (Supplementary Figure S1C).

### 2.2. Expression Analysis of STY46 in Arabidopsis thaliana Rosettes

**Figure 1:**
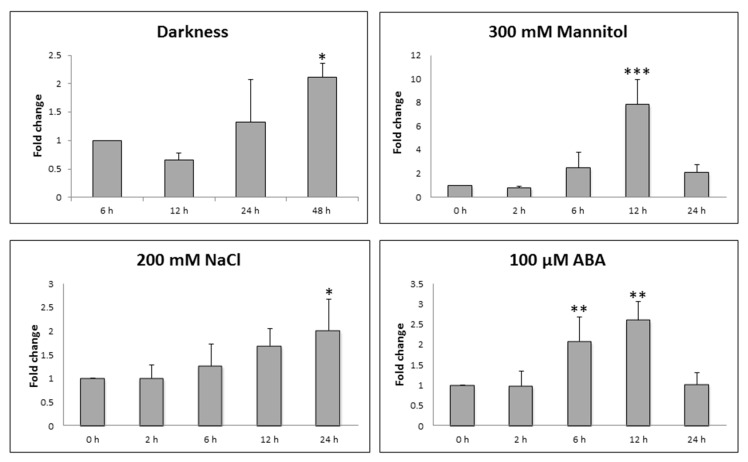
Changes in Ser/Thr/Tyr kinase isoform 46 (STY46) transcripts in wildtype plants (Col-0) under stress. The transcript level of STY46 at 0 h was set as 1. The Y-axis indicates the fold change of STY46 mRNA at different time-points compared to 0 h. Arabidopsis Col-0 plants were treated with 300 mM mannitol, 200 mM NaCl, and 100 µM ABA at 0 h (the beginning of day), 6 h (midday), 12 h (end of day), and 24 h (end of day), respectively. For darkness treatments, the Col-0 plants were exposed to 24 h extended dark (48 h). The asterisks indicate that there is statistically significant difference of transcripts level in stress-treated plants compared with 0 h (‘*’, 0.01 < *p* < 0.05; ‘**’, 0.001 < *p* < 0.01; ‘***’, 0 < *p* < 0.001).

To confirm that STY46 is responsive to sugar starvation in vivo, three-week-old *Arabidopsis thaliana* ecotype Columbia (Col-0) plants were exposed to extended darkness. The study period spanned 48 h, and included an additional 24 h dark period after the 12 h/12 h day/night photoperiod to induce a sugar deficit. Expression of STY46 in rosette was not regulated by the diurnal cycle but was induced by extended darkness (Figure 1).

The data mined from Genevestigator^®^ suggested that STY46 was responsive to abiotic stress, but there was no data for salinity or osmotic stress (the latter used as a proxy for water-deficiency), which are major factors limiting plant productivity [33]. Therefore, changes in STY46 transcript levels were measured in the rosette of three-week-old Arabidopsis exposed to 200 mM NaCl and 300 mM mannitol, conditions that our previous work indicates, trigger the most dynamic changes in the carbon flux [34]. As Figure 1 shows, the transcript level of STY46 was induced 12 h post exposure to 300 mM mannitol. Similarly, STY46 was induced 24 h after 200 mM NaCl treatment.

Since the expression of STY46 was up-regulated by sugar starvation conditions and abiotic stresses, including salinity and osmotic stress, STY46 responsiveness to abscisic acid (ABA), a hormone that is well known for its role in environmental stress response, was tested. Three-week-old Arabidopsis was exposed to ABA at a concentration of 100 µM, identical to that previously used to study a peanut STY-homologue [28]. Here, STY46 transcript levels in the rosette were up-regulated 6 h and 12 h post exposure (Figure 1).

### 2.3. Generation of STY46 Transgenic Plants

**Figure 2:**
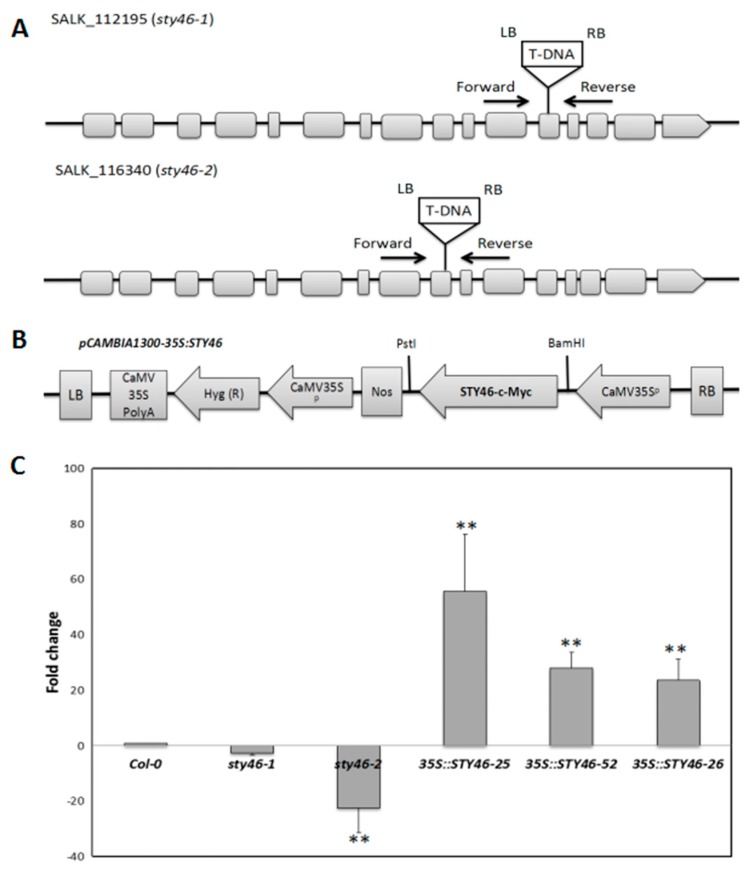
Generation of stable STY46 transgenic lines. (**A**) A gene model showing the position of the T-DNA insertion. Two stable homozygous STY46-mutant lines: STY46-1 (SALK_112195, T-DNA insertion in the 12th exon) and STY46-2 (SALK_116340, T-DNA insertion in the 9th exon). (**B**) Schematic diagram of the STY46 overexpressing construct. Left border (LB); CaMV35SPolyA: Untranslated region of CaMV 35S gene; Hyg (R): Hygromycin phosphotransferase gene that confers Hygromycin resistance; CaMV35S^p^: CaMV 35S promoter; NOS: transcriptional terminator sequence of the nopaline synthase gene; STY46-c-Myc: a c-Myc-epitope-tagged full-length cDNA of STY46; right border (RB). (**C**) qRT-PCR showing relative amounts of mRNA levels in STY46 knockout (KO), and overexpressing (OE) lines. Average qPCR data were derived from nine data measurements for each sample. Error bars represent the standard deviation. The asterisks indicate the statistically significant differences of transcripts levels between genes in the control and stress-treated plants (*n* = 3, ‘*’, 0.01 < *p* < 0.05; ‘**’, 0.001 < *p* < 0.01; ‘***’, 0 < *p* < 0.001).

To functionally test the role of STY46 in plant growth and abiotic stress response, lines with varying levels of STY46 were generated. Two stable homozygous STY46 T-DNA insertion mutant lines were obtained: STY46-1 (SALK_112195) with the insertion in the 12th exon and STY46-2 (SALK_116340) with the insertion in the 9th exon (Figure 2A). Three transgenic Arabidopsis lines, homozygous for the presence of the STY46-overexpressing construct (Figure 2B), were generated.

Leaf STY46 expression in the STY46-1 and STY46-2 mutants were 2.5-fold and 13.9-fold lower than the Col-0 control (Figure 2C). The 35S::STY46-25 genotype (called OE-25), 35S::STY46-52 (called OE-52), and 35S::STY46-26 (called OE-26), had 55-fold, 28-fold, and 23-fold higher STY46 transcripts compared with Col-0 (Figure 2C). The STY46 T-DNA insertion mutants (STY46-1, STY46-2), overexpressing lines (OE-25, OE-52, and OE-26), and Col-0 represent Arabidopsis lines with low, high, and normal STY46 expression were used in subsequent experiments.

### 2.4. Characterization of STY46 Transgenic Plants under Control Conditions

Carbon availability impacts plant growth, including cell division, cell expansion, and morphogenesis, determining growth rate, biomass production, and yield [8]. If STY46 has a role in regulating carbon availability, differences in the growth of source and sink tissues of STY46 transgenic lines with differential STY46 transcript levels would be expected.

**Figure 3:**
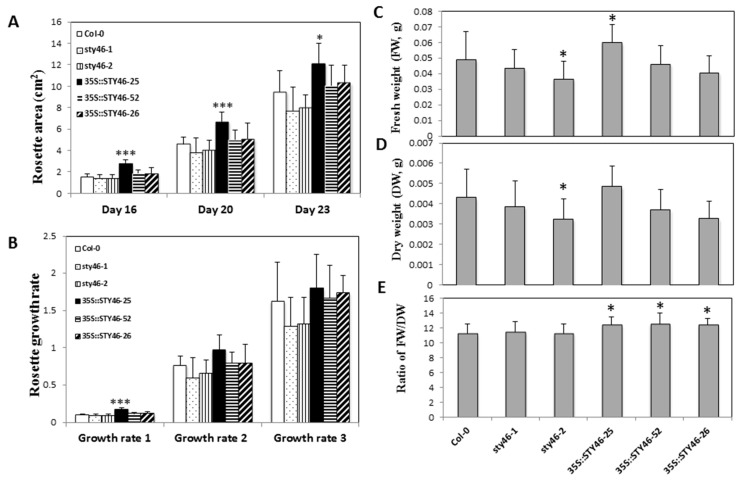
Change in rosette growth parameters. Rosette (**A**) surface area, (**B**) growth rate, (**C**) fresh weight (FW), (**D**) dry weight (DW), and (**E**) FW/DW, of STY46 transgenic genotypes. The rosette area was measured at 16 days, 20 days, and 23 days, respectively, and the growth rate during 0–16 days (growth rate 1), 16–20 days (growth rate 2), and 20–23 days (growth rate 3) was calculated. The asterisks in (**A**,**B**) indicate the statistically significant differences between transgenic lines and the wild type control (*n* = 12, ‘*’, 0.01 < *p* < 0.05; ‘**’, 0.001 < *p* < 0.01; ‘***’, 0 < *p* < 0.001). The asterisks in (**C**–**E**) indicate the statistically significant differences between transgenic lines and the wild type control (*n* = 16, *p* < 0.05).

First, to examine whether STY46 affects growth in source tissue, the rosette size of transgenic plants was measured using 16-, 20-, and 23 days-old plants, and the rosette growth rates during 0 to 16 days (growth rate 1), 16 to 20 days (growth rate 2), and 20 to 23 days (growth rate 3) were determined. Among the transgenic lines, OE-25 had a larger size, shown as a greater rosette area (82.8%, 45.5%, 27.6%) at each time-point compared to the control (Figure 3A). At the early seedling stage (day 0–16), OE-25 also had a higher rosette growth rate. However, no difference in the daily growth rate of the OE-25 rosette was detected after day 16 (Figure 3B), which suggested that the impact of STY46 on growth is more significant during the younger seedling stage.

Second, to investigate if STY46 expression affects the biomass of source tissue, rosette fresh weight, dry weight, and the fresh/dry weight ratio (FW/DW) were determined in four-week-old STY46 transgenic plants. Compared with Col-0, STY46-2, one of the STY46 mutant lines, had a lower fresh weight and dry weight, while OE-25, one of the three overexpression lines, had a higher fresh weight, but there was no difference in fresh or dry weight in the other transgenic lines (Figure 3C,D). For the FW/DW ratio, the two mutant lines was not different from Col-0; however, all three OE lines were higher than Col-0 (Figure 3E).

**Figure 4:**
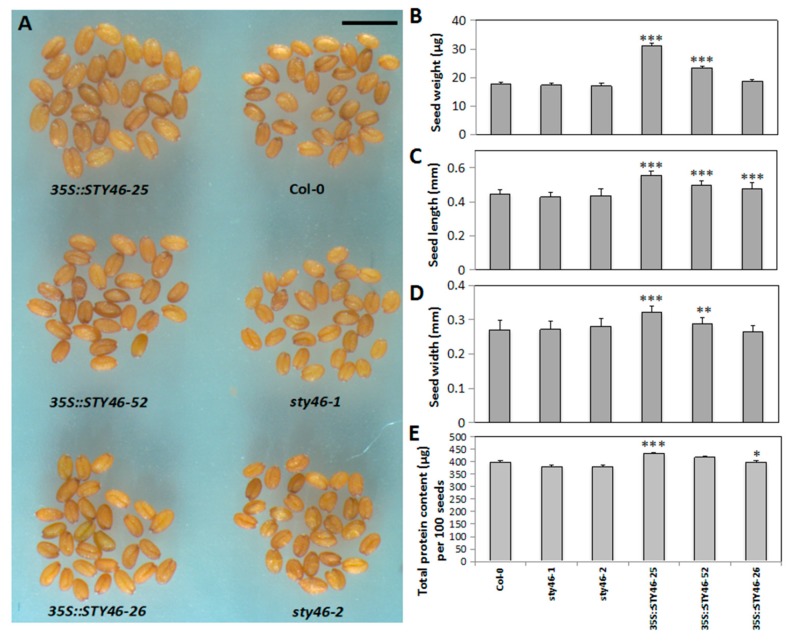
Characteristics of seeds harvested from the STY46 transgenic lines. Shown are (**A**) seeds as visualized under a microscope, and (**B**), seed length, (**C**) seed width, (**D**) seed weight and (**E**) total seed protein content The asterisks indicate statistically significant differences between transgenic lines and the wild type control. For (**A**–**C**) (*n* = 30); for (**D**,**E**) (*n* = 3) ‘*’, 0.01 < *p* < 0.05; ‘**’, 0.001 < *p* < 0.01; ‘***’, 0 < *p* < 0.001).

If STY46 is involved in the SSR, altering its expression may affect the carbon available for partitioning to the sink tissues, especially the primary sink, the seeds. Therefore, seed size and seed biomass were determined in STY46 transgenic lines. Microscopic observation of the seeds indicated that the overexpression lines were enlarged relative to the wild type (Figure 4A), and this was manifested as both increased seed length (Figure 4B) and width (Figure 4C). In agreement with the visual observation, quantitative assessments of OE-25 and OE-52 seeds showed that they were larger (*p* < 0.001) (Figure 4D) and had a higher protein content (Figure 4E) compared to the wild type control. However, the STY46 mutant lines did not differ in seed size or weight.

### 2.5. Characterization of STY46 Transgenic Plants under Stress

**Figure 5:**
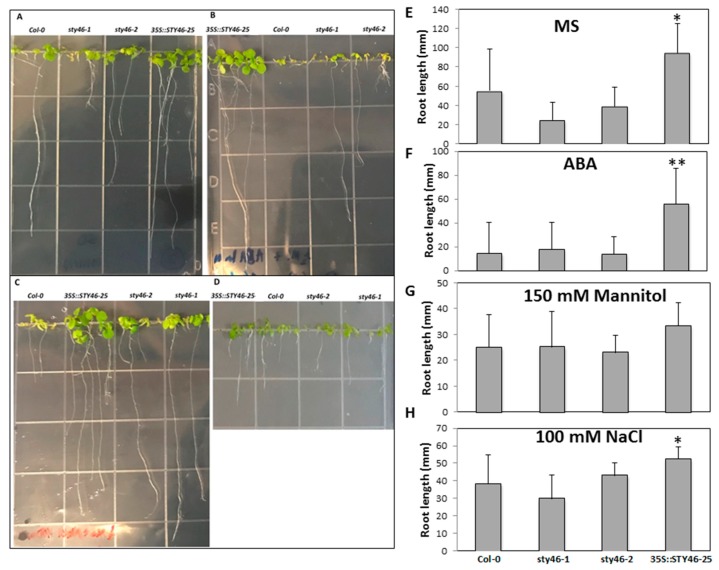
The morphology and root length of STY46 transgenic seedlings grown under stress. Wild type Col-0, STY46 mutant lines (STY46-1, STY46-2), and STY46 overexpression lines (35S:STY46-25) were germinated and grown in ½ MS medium without sucrose (**A**,**E**), ½ MS with 1 µM abscisic acid (ABA) (**B**,**F**), ½ MS medium with 100 mM NaCl (**C**,**H**), and ½ MS medium with 150 mM mannitol (**D**,**G**). Root length in seedling growing under stress. The photos were taken at 21 days old. Differences in root length were determined by quantitative analysis of 15-roots. Shown here is a random sampling of plants. The asterisks indicate statistically significant differences of root length (**E**–**H**) between transgenic lines and the wild type control (*n* = 15, ‘*’, 0.01 < *p* < 0.05; ‘**’, 0.001 < *p* < 0.01).

Since the transcript of STY46 was induced by sugar starvation conditions (Figure 1), T-DNA insertion mutants and OE lines were used to test the role of STY46 under these conditions, i.e., extended darkness, salinity, and osmolarity. Transgenic plants were also exposed to exogenous ABA to determine if this stress hormone could interact with a pathway influenced by STY46 and affect growth. Wild type Col-0 and STY46 transgenic lines (STY46-1, STY46-2, OE-25) were grown in ½ Murashige and Skoog (MS) medium without sucrose and ½ MS with 1 µM ABA, 100 mM NaCl, or 150 mM Mannitol. Among these genotypes, OE-25 showed better performance under sugar deficit (Figure 5A), 1 µM ABA (Figure 5B), and 100 mM NaCl (Figure 5C). Sugar starvation due to environmental stress is accompanied by a very rapid inhibition of root extension growth [8]; therefore, the root length of three-week-old plant exposure stress was measured. There was no significant difference between STY46 mutant lines and Col-0; however, the root length of OE-25 grown under sugar deficit, salinity, and ABA was 71.6%, 36.0%, and 287.8% longer than Col-0, respectively (Figure 5E–H). The root growth reflects that OE-25 had better ability to survive abiotic stress and a temporary carbon and energy deficit.

**Figure 6:**
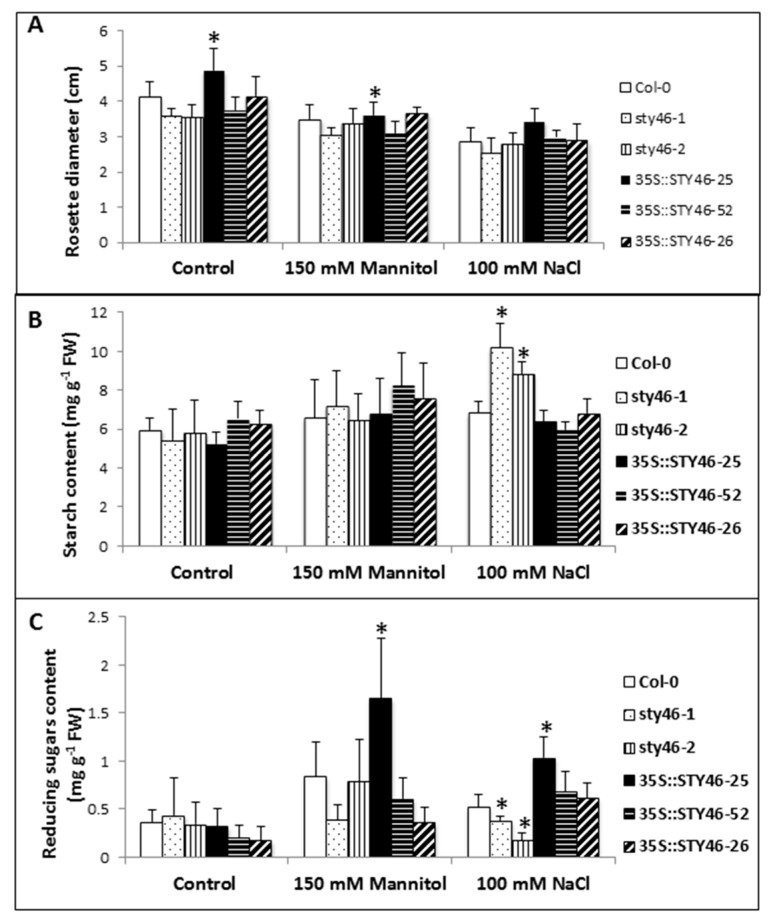
Growth and carbohydrate content of STY46 transgenic lines grown under control conditions and abiotic stress. Rosette diameter (**A**), starch content (**B**), and reducing sugar content (**C**) of two-week-old plants were exposed to 150 mM mannitol and 100 mM NaCl for 10 days. Samples for carbohydrate measurements were harvested at the end of the day. The asterisks indicate statistically significant differences between transgenic lines and the wild type control (*n* = 6, *p* < 0.05).

T-DNA insertion mutants (STY46-1, STY46-2) and OE line (OE-25, OE-52, and OE-26) response to abiotic stress was examined. Two-week-old plants were exposed to 150 mM Mannitol and 100 mM NaCl for 10 days. The rosette diameter was measured after each stress treatment and was used as an indicator of rosette size. Under the non-stressed condition, OE-25 had a significantly (*p* < 0.05) larger rosette size compared with Col-0 (Figure 6A). Under salinity stress, OE-25 rosette size was still larger compared to Col-0 (Figure 6A). However, under osmotic stress, there was no significant difference between OE-25 and Col-0 (Figure 6A). Because OE-25 rosette had a higher fresh weight (Figure 3), the decreased rosette growth might have been due to the reduced cell expansion rate during osmotic stress.

The idea that STY46 may be part of a biological pathway integrating carbon availability and abiotic stress was directly tested. The STY46 genotypes were exposed to 150 mM mannitol and 100 mM NaCl stress, and carbohydrates were assayed in rosettes and harvested at the end of the light period, when carbohydrate content was highest [34]. There were no differences in starch and reducing sugar contents among genotypes under non-stressed conditions (Figure 6B,C). However, after ten days of exposure to 100 mM NaCl, the STY46 mutant lines (STY46-1, STY46-2) had higher starch, but decreased reducing sugar contents, respectively (*p* < 0.05). The three STY46 overexpressing lines had no changes in starch content, but OE-25 leaves had higher reducing sugars. Ten days after 150 mM mannitol treatment, there was no difference in starch content (Figure 6B) among the transgenic lines; however, OE-25 showed significantly (*p* < 0.05) higher reducing sugar content (Figure 6C).

**Figure 7:**
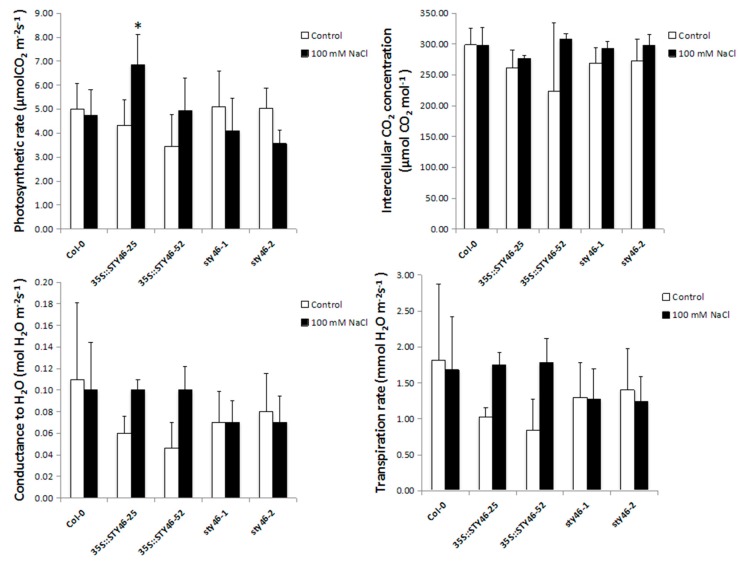
Photosynthetic performance of STY46 transgenic lines under control conditions and salinity stress. The transgenic lines were exposed to 100 mM NaCl for one week and the photosynthetic rate, water conductance, intercellular CO_2_ concentration, and transpiration rate were measured. The asterisks indicate statistically significant differences between transgenic lines and the wild type control (*n* = 5, *p* < 0.05).

STY46 regulates the chloroplast pre-protein import and photosynthetic capacity in deetiolated seedlings [25]. This knowledge, plus our observations of a better response of OE-25 under stress, opens the possibility that in adult plants, STY46 may alter plant photosynthetic capacity under stress. We chose to expose OE-25 plants to salinity stress because there was a clear carbohydrate phenotype and better growth response under this condition, compared to the control. The transgenic lines were exposed to 100 mM NaCl for one week, and various indicators of photosynthetic performance were measured. Compared with the control, OE-25 had a higher photosynthetic rate under 100 mM NaCl stress. There were no changes in water conductance, intercellular CO_2_ concentration, or transpiration rates (Figure 7), suggesting that the changes in photosynthetic rate were not from alterations in stomatal conductance, but more likely from changes in the photosynthetic capacity of the chloroplasts.

### 2.6. ^14^CO_2_ Partitioning and Allocation in Stress-Treated Plant Tissues

**Figure 8:**
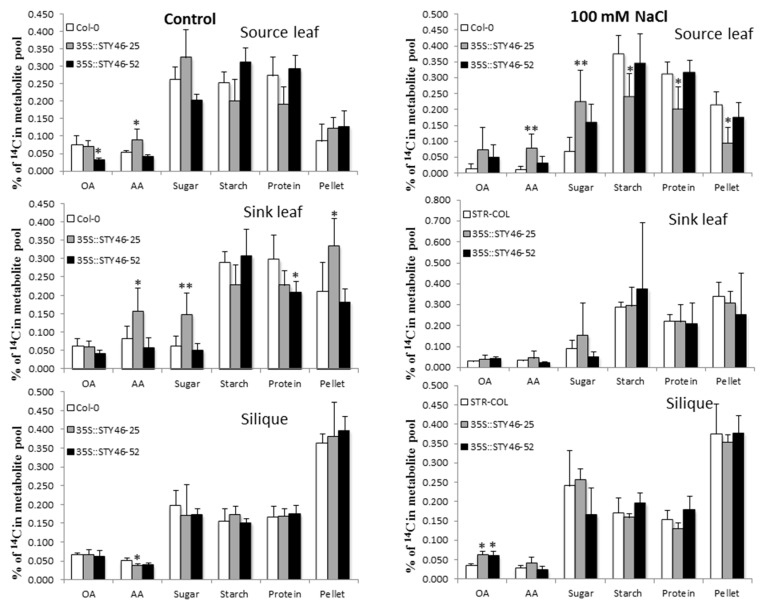
^14^C partitioning in Arabidopsis source and sink tissues under normal and salinity stress. The incorporation of ^14^C into sugars, starch, amino acids (AA), protein, organic acids (OA), and pellets in the source leaf, sink leaves, and silique at midday was determined. The total label in each tissue was set to 100%. The asterisks indicate a significant difference between the control and salt-treated plants (*n* = 5, *p* < 0.05).

Carbon partitioning into major metabolite pools changes dynamically in response to various abiotic stresses [34]. To further investigate if STY46 could regulate carbon partitioning and allocation under abiotic stress, four-week-old Arabidopsis seedlings were exposed to 100 mM NaCl for one week, and a single mature source leaf was fed with ^14^CO_2_ at the beginning of the day for 30 min, as previously described [34]. The labeled source leaf, unlabeled sink leaves, and the silique were harvested separately at the middle of the day. Within each tissue, the incorporation of ^14^C into the main metabolite pools (sugars, amino acids, organic acids, starch, protein, and the remaining insoluble compounds (RICs)) was determined. Under control conditions, carbon partitioning in source leaf was the same across genotypes. However, in the sink leaf and siliques, more ^14^C was partitioned into amino acids and sugars in OE-25 compared to the Col-0. Further, when the plants were exposed to 100 mM NaCl, ^14^C partitioning into amino acids and sugar in OE-25 was amplified, while less was diverted into starch and protein (Figure 8).

## 3. Discussion

The aim of this work was to test a potential role for STY46 in plant growth and abiotic stress as part of an integrated response to reduced carbon availability. Transgenic Arabidopsis lines with varying transcript levels of STY46 were developed. The impact of STY46 on growth in source (rosette) and sink (seed) tissue of these transgenic lines with varying levels of STY46 transcripts was evaluated under control conditions. Further, the role of STY46 in regulating carbon use under abiotic stress was examined.

### 3.1. Expression Analysis of STY46 under Stressed Conditions

The expression of STY46 was up-regulated in response to salt and osmotic stress (Figure 1). The activation was detected 12 to 24 h after exposure, which indicates that STY46 may participate in the adaptive process to stress conditions instead of functioning in an initial response to these adverse environments. STY46 was also induced by ABA (Figure 1), a key regulator of multiple environmental stress response [35]. Stress response in plants can be divided into two pathways: ABA-dependent and ABA–independent [36]. A closely-related STY46 orthologue in peanut cotyledon was not altered after two days of exposure to 100 µM ABA, which suggested that it may be part of an ABA-independent salt-signaling pathway [28]. However, in our study, the STY46 transcript was up-regulated by 100 µM ABA 6 h after exposure, earlier than the salt and osmotic response, which suggests that STY46 might be involved in an ABA-dependent osmotic and salt signaling pathway.

### 3.2. STY46 Has a Role in Regulating Growth of Arabidopsis Source and Sink Tissues

In most experiments performed in this study, the STY46 T-DNA mutant lines, STY46-1 and STY46-2, did not show a significant phenotype compared to the wild-type Col-0. This is likely due to gene redundancy, since there are 57 isoforms in the STY family, such as STY8 and STY17, that may have overlapping functions [25]. Interestingly, these isoforms did not appear to be regulated similarly to STY46 in the SSR gene co-expression network. Among the three STY46 OE lines, OE-25 showed a more contrasting phenotype, which might be due to the very high STY46 transcript level in the OE lines. As shown in Figure 2C, OE-25, OE-52, and OE-26 had 55.7-, 28.1-, and 23.6-fold higher STY46 transcript relative to Col-0, respectively; therefore, STY46 transcripts had to be dramatically high before an effect could be detected. A Pearson’s correlative analysis of the data showed that STY46 transcript levels changed in synchrony with seed protein content (R^2^ = 0.868, *p* < 0.05). There were also weak but significant correlations between STY46 expression and water conductance under control conditions (R^2^ = 0.230, *p* < 0.05) and intercellular CO_2_ concentration under 100 mM NaCl (R^2^ = 0.119, *p* < 0.05). Furthermore, the correlation of changes in root growth on MS media (R^2^ = 0.836) and root growth under salinity (R^2^ = 0.655) with the STY46 transcript were suggestive (*p* = 0.051 and *p* = 0.064, respectively). Correlation is not causation, but in the context of the hypothesis tested, it provides support to the theory that STY46 may have a role in plant response to some stresses.

Under control conditions, OE-25 had a larger rosette size (Figure 3) and higher rosette growth rate during the early seedling stage (Figure 3), which suggested that STY46 at high levels of expression influences rosette growth. There were no changes in rosette growth rate during days 16–23, which suggests that the effect of STY46 was significant in younger, but not in adult tissues. This is consistent with a previous study, which suggested that STY46 function is more pronounced during developmental stages that require a massive influx of preprotein into chloroplast, e.g., cell differentiation and expansion in leaves, rather than stages associated with growth maintenance, e.g., adult leaf tissue [25].

Interestingly, all three OE lines showed a higher FW/DW ratio compared to Col-0 (Figure 3). Therefore, STY46 might affect relative water content or dry matter content in rosette. This could also be due to endoreduplication or somaclonal variation due to transgenesis [37]. Whether STY46 increases the relative water content of the entire plant or only in source tissues needs to be further studied.

During the early stages of embryo development, a higher amount of sugars are transported from the phloem to supply seeds with the carbon needed for growth, which occurs through rapid cell division and cell enlargement [38]. During the maturation stage, cell division ceases and lipids and protein are deposited [38] and are responsible for seed dry weight [39,40]. Therefore, the increased seed size and dry weight (Figure 4) in OE-25 and OE-52 indicate that more carbon was imported into the seed during early embryo growth compared to Col-0. Genevestigator analysis showed that STY46 is highly up-regulated in chalazal endosperm, which connects the seed to the maternal tissue [41,42] and regulates resource uptake from the parent into the developing seed [39,43]. These data, and the results presented here, collectively suggest that STY46 might have a role in regulating carbon resource accumulation in seed, but this would need to be directly tested.

### 3.3. The Role of STY46 in Abiotic Stress Response

The up-regulation of STY46 expression (Figure 1), together with the better growth performance of OE-25 under abiotic stress and ABA treatment (Figure 5), suggest that STY46 has a positive role in plant abiotic stress response and this response might be mediated by the ABA signaling pathway. A previous study showed that the SnRK2.3 transcript was down-regulated in STY46STY8STY17 triple mutants [25]. SnRK2.3 is a protein kinase that mediates the ABA signaling pathway to regulate seed germination, root growth, seedling growth, and proline accumulation [44]. Whether the involvement of STY46 in the ABA signaling pathway is mediated by SnRK2.3, and how STY46 interacts with SnRK2.3, needs further investigation. Genevestigator analysis showed that STY46 expression closely correlates with that of *AT1g23870*, a gene encoding trehalose-phosphatase synthase 9, which is involved in trehalose-6-phosphate (T6P) metabolism (Supplementary Figure S2A). STY46 was also induced in Arabidopsis mesophyll protoplasts transiently expressing At3g01090, a gene encoding SnRK1.1, which is responsive to sugar starvation (Supplementary Figure S2B). Therefore, STY46 might be involved in the T6P/SnRK1 pathway and deserves further study.

Changes in carbohydrate metabolism under abiotic stress depend on many factors, such as the duration and intensity of different stresses, the different tissue types, and the tissue developmental stage [6]. Under osmotic and salinity stress and the overexpression line, OE-25 accumulated more sugars in the rosette. In contrast, the mutant lines (STY46-1, STY46-2) had decreased sugar content under salinity stress (Figure 6C). The accumulation of sugars is one mechanism of plant response to environment stress, primarily by acting as compatible solutes to protect cellular membranes and proteins under stress [6,45,46] and as reactive oxygen species scavengers [47]. The accumulated reducing sugars may also act as sources of energy and as carbon building blocks, directly through the respiratory cycle for the biosynthesis of metabolites necessary for stress response [20]. Changes in sugar levels under stress can be sensed by stress signaling pathways and lead to the activation of genes involved in a stress response [48,49].

Under salinity stress, the carbohydrate content in the rosettes, at the end of the day, differed in the mutants compared to OE-25. Starch content in OE-25 was the same as the control, but there was an increase in sugars, whereas in the mutant lines, there was decreased sugar but higher starch content (Figure 6). The lower sugar and higher starch content mutants might be due to defective starch degradation caused by the loss of STY46 expression. Starch can act as a “sugar-source” when carbon is deficient [50], and, under environmental stress, starch metabolism is often regulated to supply optimal sugar levels necessary for stress response [6]. Previous studies in Arabidopsis showed that 150 mM salt stress results in increased sugar accumulation and reduced starch content [51], highlighting that STY46, is necessary for this physiological process. Our work suggests the activation of a regulatory mechanism, whereby carbon is preferentially partitioned into osmoprotectants (sugars, amino acids, organic acids) at the expense of storage compounds (starch or protein) as a positive way to respond to salinity stress [34]. The ^14^CO_2_ labeling experiment reinforced this and showed that OE-25 partitioned more ^14^C into amino acids (AA) and sugars, while it partitioned less into starch and protein in source leaf, which suggested that OE-25 is more responsive to salinity stress than the wild type.

## 4. Materials and Methods

### 4.1. Analysis of STY46 T-DNA Mutant Lines

Seeds of *Arabidopsis thaliana* Columbia-0 ecotype (wild type) and two independent T-DNA insertion mutants of STY46 (SALK_112195: sty46-1, SALK_116340: sty46-2) were obtained from the Arabidopsis Information Resource (Ohio State University, Columbus, OH, USA). T-DNA mutant lines were screened and self-pollinated to homozygosity and tested by PCR. Primers used are listed in Supplementary Table S1.

### 4.2. Generation of STY46 Overexpressing Transgenic Arabidopsis Lines

A myc-epitope-tagged STY46 (GenBank Accession: NM_120008.2) full-length cDNA was amplified from the cDNA library of Arabidopsis Columbia ecotype (Col-0) rosette as a *Bam*HI/*Pst*I fragment, using the primers in Supplementary Table S2. The STY46 PCR fragment was digested and cloned to the multiple cloning sites located between the 35S Cauliflower mosaic virus (CaMV) and the NOS terminator of the *p*CAMBIA1300 (Center for the Application of Molecular Biology of International Agriculture, Canberra, Australia). The recombinant construct the pCAMBIA1300 was electroporated (Gene Pulser, BIO-RAD, Hercules, CA, USA) into Agrobacterium tumefaciens strain EHA105. Positive transformants were selected on left border (LB) agar plates, supplemented with 50 μg/mL kanamycin sulfate (Sigma-Aldrich, St. Louis, MO, USA) and 10 μg/mL Rifampicin (Sigma-Aldrich, St. Louis, MO, USA) and double-checked by restriction enzyme digestion. Agrobacterium tumefaciens cell harbor transformation constructs were cultured and resuspended into 5% (w/v) sucrose solution, containing 0.05% (v/v) Silwet L-77. Developing inflorescences of Arabidopsis Col-0 were dipped in the cell suspension for 5 s. Seeds from transformed Arabidopsis were selected using the MS media, supplemented with 2 mg/L hygromycin B (Sigma-Aldrich, St. Louis, MO, USA). The transgenic lines were self-pollinated and genotyped until stable lines homozygous for the construct were identified (T_3_). The levels of STY46 transcripts were compared between the transgenic plants transformed with STY46 and Col-0 using a quantitative real-time PCR approach with primers listed in Table S3.

### 4.3. Stress Treatments

For gene expression analysis: Col-0 was grown on a ½ Murashige and Skoog (½ MS) agar medium (½ MS salts, 1% (w/v) agar, 1% (w/v) sucrose, pH 5.7). Three-week-old seedlings were taken out from the medium and submerged into Hoagland solution [52] (12 h/12 h day/night, 21/23 °C), containing 300 mM mannitol, 200 mM NaCl, or 100 µM ABA for 0, 2, 6, 12, and 24 h. For darkness treatment, the Col-0 plants were exposed to 24 h of extended dark (48 h). Rosettes were harvested from each plant.

Characterization of transgenic lines, Col-0 and STY46 transgenic seeds, were initially sterilized and grown vertically in a 9 × 9 cm petri dish with ½ MS, containing either 100 mM NaCl, 150 mM mannitol, or 1 μM ABA. Three replicate plates were used for each treatment. Plants were also grown in soil in a growth chamber at 21 °C with 16 h/8h day/night, 150 μmol photon m^−2^ s^−1^ light intensity, and 60% relative humidity. Two-week-old plants were irrigated with nutrient solution, containing either 150 mM mannitol or 100 mM NaCl.

### 4.4. Genomic DNA Extraction

A genomic DNA extraction method was modified based on a previous study [53]. Two young Arabidopsis leaves were harvested into 2 mL Eppendorf tubes and ground into a powder with liquid nitrogen. Approximately 500 μL of DNA extraction buffer (prewarmed to 65 °C) was added and mixed with 500 μL of chloroform: isoamyl alcohol, 24:1 (v/v). The homogenate was centrifuged for 10 min at 13,500× g and 400 μL of the supernatant was transferred to a new sterile 1.5 mL tube. Approximately 400 μL of isopropanol was added then centrifuged at 13,500× g for 10 min. The pellet was washed with 100 μL of 70% (v/v) ethanol, air-dried, and then resuspended in 50 μL ddH_2_O.

### 4.5. Quantitative Real-Time Reverse Transcript-PCR

RNA extraction was done as described [34]. Arabidopsis tissue (100 mg) was ground into a fine powder with liquid nitrogen in a 2 mL Eppendorf tube, and 1 mL TRIzol^®^ reagent (Invitrogen, Carlsbad, CA, USA) was added. cDNA was synthesized using the High Capacity cDNA Reverse Transcription Kit (Applied Biosystems, Vilnius, Lithuania). *Actin2* was used as the reference to normalize gene expression. The primers for gene amplification are shown in Supplementary Table S3. Primers were optimized and the efficiency was determined by making a standard curve with different dilutions of cDNA. The ddCt method [54] was used to analyze the expression of each gene based on the fold-changes of transcripts in the experimental sample compared with the control sample. All procedures were as previously described [34].

### 4.6. Carbohydrate Analysis

Approximately 100 mg of rosettes from each plant was ground to a fine powder and boiled in 1 mL 80% (v/v) ethanol for 10 min and centrifuged, and the supernatant was poured into a separate 4 mL tube. This process was repeated twice more; each time, the supernatant was pooled, and the pellet was kept for starch measurement. Assay of starch and sugars followed our previously used procedure [34].

### 4.7. ^14^CO_2_ Pulse-Chase Labeling and Fractionation of ^14^CO_2_-Labelled Plant Tissue

The ^14^CO_2_ feeding was carried out on the 8th mature leaf of each plant after stress-treatment or non-treatment control in the “leaf chamber” at the beginning of the day for 30 min. ^14^CO_2_ was generated from 0.08 MBq NaH_14_CO_3_ and acidified with 200 μL of 10% (v/v) lactic acid in the reservoir chamber. The generated ^14^CO_2_ was pumped into a leaf chamber via the tubing system, where a single leaf of a plant was exposed to ^14^CO_2_. After each 5 min pulse, the stopcocks were turned and the new plants were replaced successively. At the end of the feeding, 500 μL of 10% (v/v) potassium hydroxide was used to stop ^14^CO_2_ generation and capture the residue ^14^CO_2_ in the chamber. The labeled leaf, unlabeled leaves, and siliques of each plant were harvested separately. Each sample was homogenized in liquid nitrogen, boiled for 10 min in successive 80%, 50%, and 20% (v/v) ethanol, and then separated into the soluble and insoluble fractions by centrifugation. The ^14^C in each fraction (organic acids, amino acids, sugars, starch, protein, and cell wall) was measured, following our previously used procedure [34].

### 4.8. Photosynthesis Measurements

Photosynthesis was measured following the method previously used [55]. An Arabidopsis leaf was placed inside a controlled-environment chamber using a Li-6400 portable gas-exchange system (Li-6400, Li-Cor, Inc., Lincoln, NE, USA) with saturating light (1200 μmol·m^−2^ s^−1^) and 400 μmol·mol^−1^ CO_2_. The temperature was set to 28 °C.

### 4.9. Assay of Rosette Size and Biomass

Col-0, STY46 T-DNA insertion mutants, and OE lines were grown in a growth chamber with conditions described in Section 4.3. The rosette of each plant was photographed at day 16, day 20. and day 23, and the rosette area was determined by ImageJ [56]. The rosette growth rates during 0 to 16 days (growth rate 1), 16 to 20 days (growth rate 2), and 20 to 23 days (growth rate 3) were determined by calculating the increase in the rosette area. When plants were four weeks old, the rosettes of each plant were harvested, weighed, and dried in an 80 °C oven for 2 weeks. From these, tissues, dry weight measurements, and the fresh/dry weight ratio was determined.

### 4.10. Assay of Seed Size and Seed Weight

Approximately 30 Arabidopsis seeds were fixed on the microscope slide with transparent tape. The slide was viewed with a microscope and photographed. Seed length and width was measured using ImageJ analysis software (https://imagej.nih.gov/ij/index.html), according to the instructions. For seed weight measurement, 100 Arabidopsis seeds were weighed, and each genotype was repeated three times.

### 4.11. Total Protein Extraction and Quantification

Protein content was assayed as described [57]. A total of 20 seeds (three biological replicates) were ground into a fine powder, homogenized in 250 μL acetone, and centrifuged at 16,000× g for 10 min. The vacuum-dried pellet was resuspended in 250 μL of extraction buffer, containing 50 mM Tris-HCl pH 8.0, 250 mM NaCl, 1 mM ethylenediaminetetraacetic acid, and 1% (w/v) sodium dodecyl sulfate, and then incubated for 2 h at 25 °C, before it was centrifuged at 16,000× g for 5 min. Approximately 100 μL of the supernatant was used for protein measurements using the Bradford Method Protein Assay Kit (VWR Life Science AMRESCO, Solon, OH, USA), following the manufacturer’s instructions.

### 4.12. Statistical Analysis

All tests for significant differences between transgenic lines and Col-0 control data were done using one-way ANOVA in the R environment. Public STY46 gene expression data were generated using an online expression data exploration platform Genevestigator^®^ [32]. Pearson’s Correlation analysis was performed in Microsoft Excel.

## 5. Conclusions

The present study showed that STY46, a hub gene in the SSR network, has a role in plant growth and abiotic stress response via regulating carbon use. STY46 is induced by ABA and abiotic stress. Under control conditions, the source and sink tissues of a transgenic line with high levels of STY46 transcripts accumulated more biomass compared to the wild type under osmotic and salinity stress, and carbohydrate metabolism was altered. We propose a model for STY46 in plants based on published and current data. STY46 is able to phosphorylate pre-proteins targeted to both the chloroplast [25] and the mitochondria [31] and can bind amino acid ligands during chloroplast biogenesis [58], thus helping to translocate proteins to the chloroplast and mitochondria. STY46 is thus part of integral carbon and energy generation processes that support emerging seedling transition from autotrophy to heterotrophy and, in adult plants, influence carbon and energy use via carbohydrate metabolism under stress. These roles may be performed collaboratively with other STY kinases and are highly regulated, because neither severe reductions nor moderate increases in STY46 cause visible changes in the phenotype. Although STY46 is likely to have a multiplicity of roles in the plant lifecycle, understanding how STY46 specifically connects plant stress signaling pathways to plant carbon use is important, because of the consequences for plant growth, development, and production under non-ideal conditions.

## Supplementary Materials

The following are available online at https://www.mdpi.com/2223-7747/9/1/57/s1. Sheet 1: Correlation analysis of STY46 transcripts and characterized phenotypes. The following are available online in the Supplementary Materials. Table S1: Primer sequences for T-DNA verification. Table S2: Primer sequences and PCR product used for STY46 cloning. Table S3: Primers used for quantitative RT-PCR. Figure S1: Public expression data of STY46 created from Genevestigator®. Figure S2: Public expression data of STY46 and related genes created from Genevestigator^®^.
