# Supplementary material for: A Cytosolic Protein Kinase STY46 in Arabidopsis thaliana Is Involved in Plant Growth and Abiotic Stress Response
Source: Plants (Basel). 2020 Jan 2;9(1):57. doi: 10.3390/plants9010057 (PMC7020404; doi:10.3390/plants9010057)
Supplement: Supplementary file 1 [file plants-09-00057-s001.zip › Supplementary Data Files/plants-659425-supplementary.docx]

Supplementary Material

A Cytosolic Protein Kinase STY46 in *Arabidopsis thaliana* is Involved in Plant Growth and Abiotic Stress Response

Shaoyun Dong ^1,†^, Fenglan Zhang ^2^ and Diane M. Beckles ^1,^*

^1^ Department of Plant Sciences, University of California, One Shields Avenue, Davis, CA 95616, USA; dongshaoyun@caas.cn

^2^ College of Agronomy, Inner Mongolia Agricultural University, Hohhot, 010019, China; zhangfenglan041105@imau.edu.cn

**^†^** Current address: Institute of Flowers & Vegetables, Chinese Academy of Agricultural Sciences, Beijing, China

***** Correspondence: dmbeckles@ucdavis.edu; Tel.: +01-530-754-4779

List of Supplementary Material

**Table S1.** Primer sequences for T-DNA verification.

**Table S2.** Primer sequences and PCR product used for STY46 cloning.

**Table S3.** Primers used for quantitative RT-PCR.

**Table 1.** Primer sequences for T-DNA verification.

| **PCR Product** | **Germplasm Name** | **LP Primer Sequence (5’-3’)** | **RP Primer Sequence (5’-3’)** | **BP Primer Sequence (5’-3’)** |
| --- | --- | --- | --- | --- |
| PCR amplification for T-DNA verification | SALK_112195C  (*sty46-1*) | TTGGCTATCTCTTGAAGCTGC | AAGCAAAAGGGCGTCTTTAAG | ATTTTGCCGATTTCGGAAC |
| PCR amplification for T-DNA verification | SALK_116340  (*sty46-2*) | GTCTGCAACCTTAACCACCTG | CAATCCTTCTCTCCGGAAAAG | ATTTTGCCGATTTCGGAAC |

**Table 2.** Primer sequences and PCR product used for STY46 cloning.

| **PCR Product** | **Accession ID** | **Forward Primer Sequence (5’-3’)** | **Reverse Primer Sequence (5’-3’)** | **Fragment Length (bp)** |
| --- | --- | --- | --- | --- |
| Fragment for constitutive overexpressing construct | NM_120008.2 | CGCGGATCCATGGTGATGGAGGACAACGAGAGT (*BamH*I site underlined) | AAAACTGCAGCTA***CAGATCTTCTTCAGAAATAAGTTTTTGTTC*** ATGATGTGTGGTGCTTCTCCTC (*PstI site* underlined, myc tag bolded) | 1,777 |

**Table 3.** Primers used for quantitative RT-PCR.

| **qRT-PCR amplification product** | **Forward primer sequence (5’-3’)** | **Reverse primer sequence (5’-3’)** |
| --- | --- | --- |
| *STY46* | NM_001342499.1: 1627-1646 bp  AGGTTGCAGACTTTGGGGTG | NM_001342499.1: 2052-2073 bp  TCCCTCTTCTCCTACCTCCTTG |
| *Actin2* | GGTGATGGTGTGTCT | ACTGAGCACAATGTTAC |

A


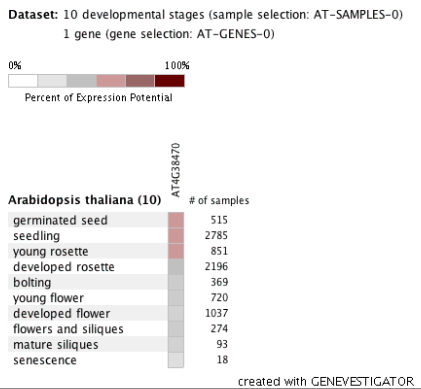


B


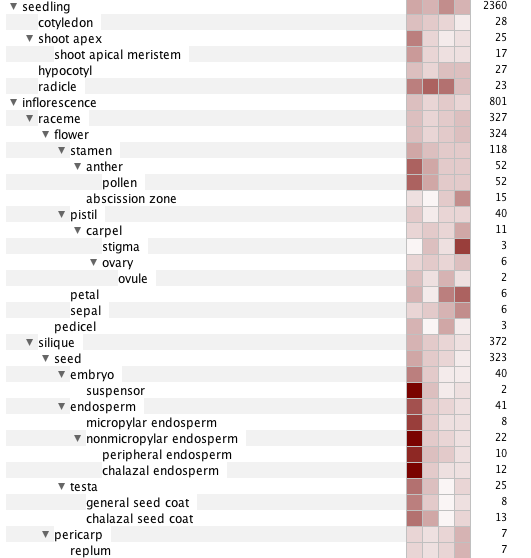

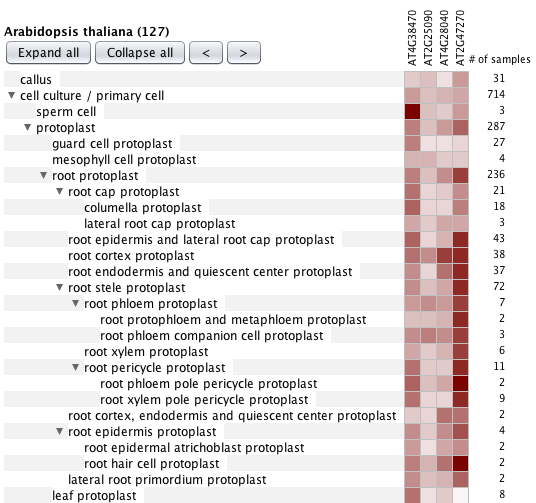


C


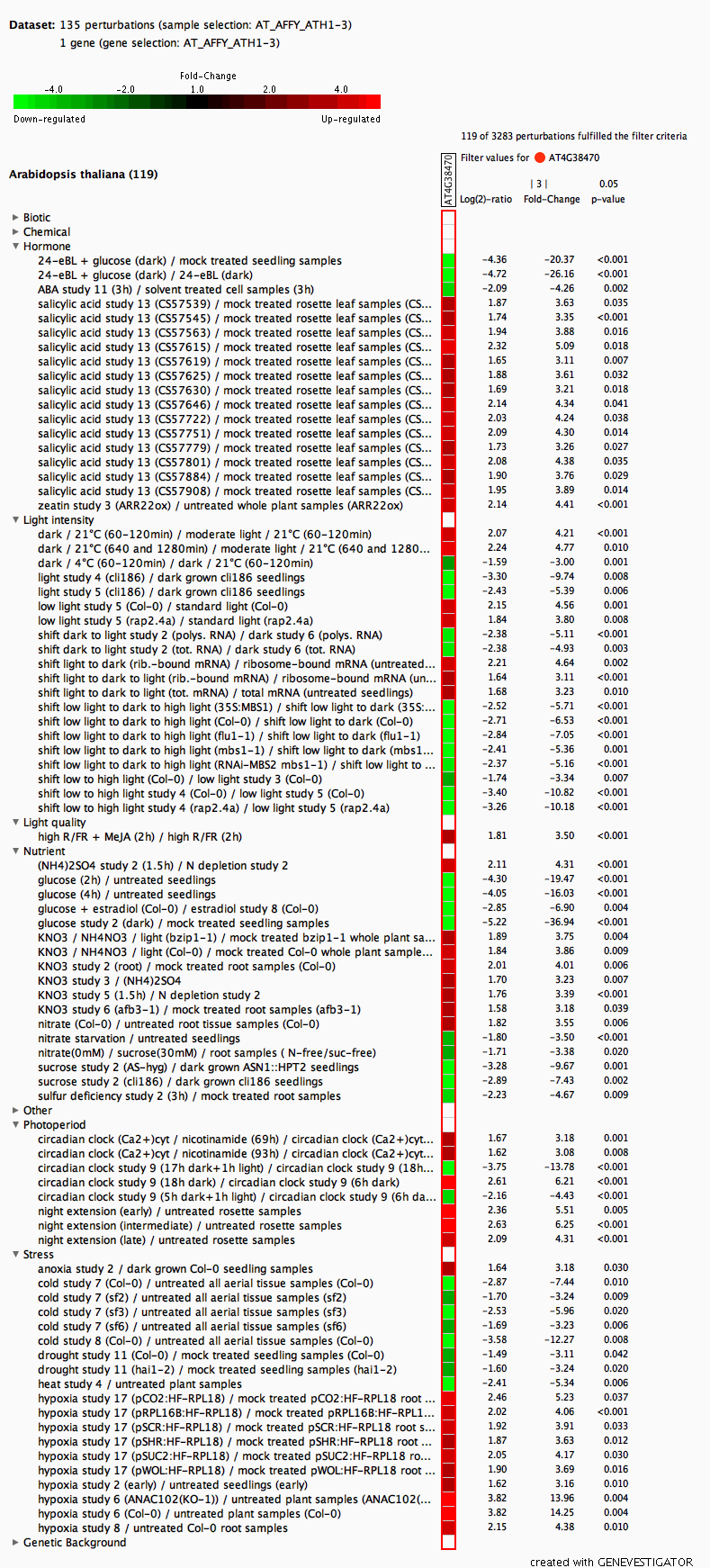


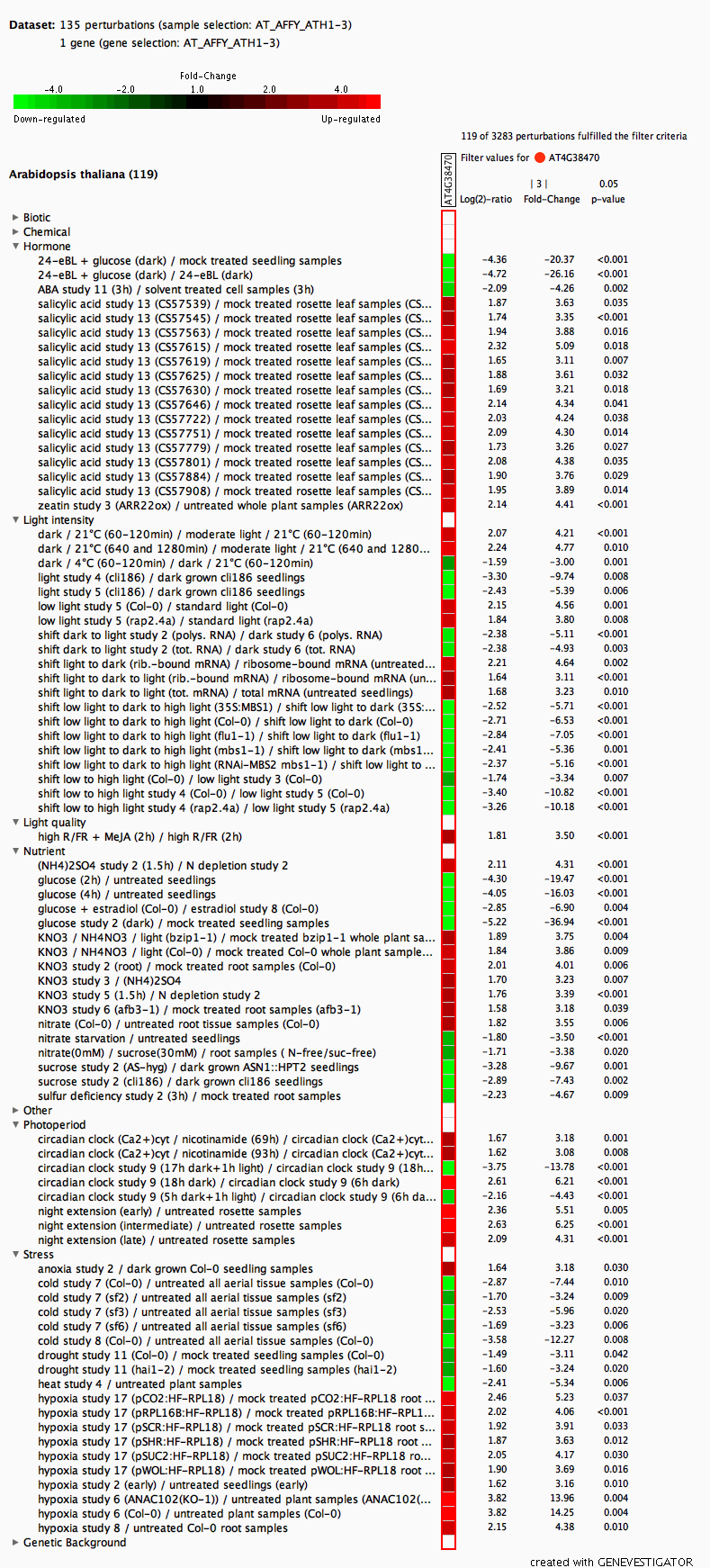


**Figure S1. Public expression data of STY46 created from Genevestigator^®^ A**. Percent of the expression potential of STY46 at different developmental stages. **B**. different tissues. **C**. and under specific conditions (fold change > 3, *p* < 0.05).

A


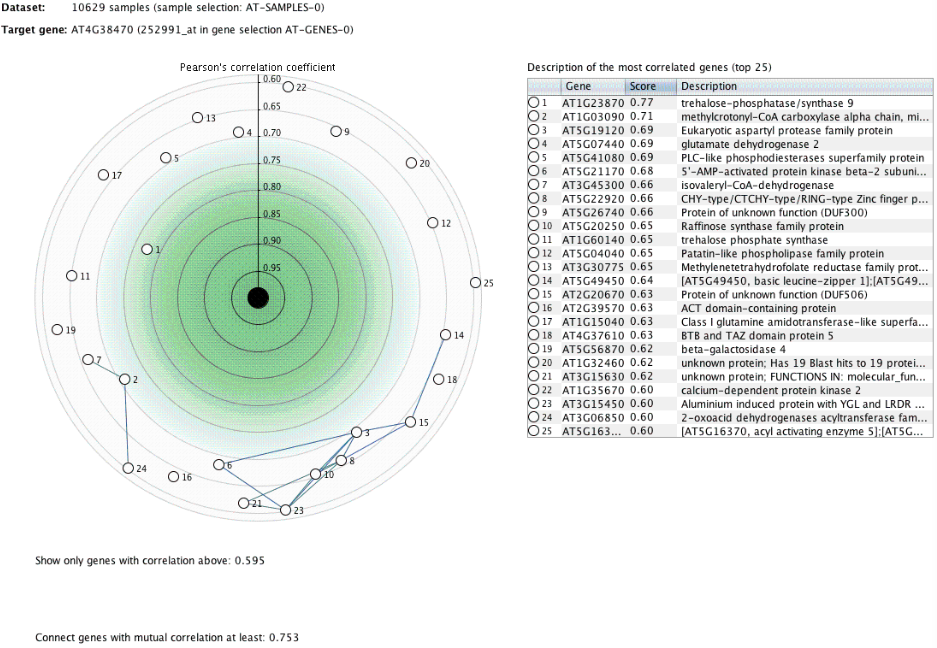


B


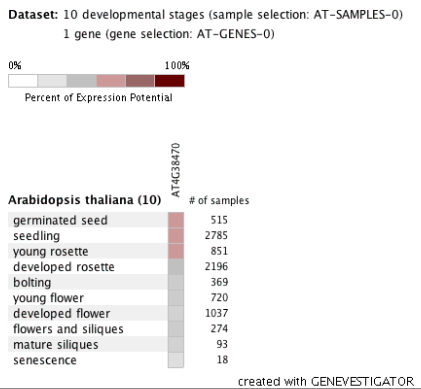


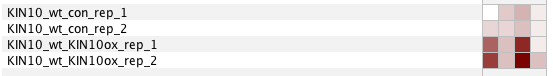


**Figure S2. Public expression data of STY46 and related genes created from Genevestigator®**. **A**. Genes most correlated with STY46. **B**. STY46 is induced in Arabidopsis mesophyll protoplasts transiently expressing KIN10 (At3g01090).
